# The assessment of CD146-based cell sorting and telomere length analysis for establishing the identity of mesenchymal stem cells in human umbilical cord

**DOI:** 10.12688/f1000research.4260.2

**Published:** 2014-08-27

**Authors:** Dimitrios Kouroupis, Sarah M. Churchman, Dennis McGonagle, Elena A. Jones

**Affiliations:** 1 Department of Biomedical Research, Foundation for Research and Technology-Hellas, Institute of Molecular Biology and Biotechnology, University of Ioannina, Ioannina, 45110, Greece; 2 Leeds Institute of Rheumatic and Musculoskeletal Medicine, University of Leeds, Leeds, LS97TF, UK; 3 NIHR Leeds Musculoskeletal Biomedical Research Unit, Leeds Teaching Hospitals NHS Trust, Leeds, LS97TF, UK

## Abstract

Adult stem cells are characterised by longer telomeres compared to mature cells from the same tissue. In this study, candidate CD146 ^+^ umbilical cord (UC) mesenchymal stem cells (MSCs) were purified by cell sorting from UC tissue digests and their telomere lengths were measured in comparison to donor-matched CD146-negative fraction.

UC tissue fragments were enzymatically treated with collagenase and the cells were used for cell sorting, colony-forming fibroblast (CFU-F) assay or for long-term MSC cultivation. Telomere lengths were measured by qPCR in both culture-expanded MSCs and candidate native UC MSCs. Immunohistochemistry was undertaken to study the topography of CD146 ^+^ cells.

Culture-expanded UC MSCs had a stable expression of CD73, CD90 and CD105, whereas CD146 declined in later passages which correlated with the shortening of telomeres in the same cultures. In five out of seven donors, telomeres in candidate native UC MSCs (CD45 ^-^CD235α ^-^CD31 ^-^CD146 ^+^) were longer compared to donor-matched CD146 ^-^ population (CD45 ^-^CD235α ^-^CD31 ^-^CD146 ^-^). The frequency of CD45 ^-^CD235α ^-^CD31 ^-^CD146 ^+^ cells measured by flow cytometry was ~1000-fold above that of CFU-Fs (means 10.4% and 0.01%, respectively). CD146 ^+^ cells were also abundant *in situ* having a broad topography including high levels of positivity in muscle areas in addition to vessels.

Although qPCR-based telomere length analysis in sorted populations could be limited in its sensitivity, very high frequency of CD146 ^+^ cells in UC tissue suggests that CD146 expression alone is unlikely to be sufficient to identify and purify native MSCs from the UC tissue.

## Introduction

Perinatal mesenchymal stem cell (MSC) sources are attracting increasing attention as an alternative to ‘gold standard’ bone marrow (BM) MSCs. Owing to its universal accessibility, umbilical cord (UC) tissue represents an attractive source of MSCs for cell therapy ^1^. However, the potency of standard culture-expanded UC MSCs, especially towards osteoblasts, chondrocytes and adipocytes, is lower compared to BM MSCs ^2, 3^. This can be explained by the fact that conventional UC MSC cultures arise from diverse clonal populations that possess varying degrees of self-renewal leading to mixed cultures that gradually lose their MSC properties ^4^. The therapeutic advantages of minimally-expanded UC MSCs would therefore include a better preservation of their native functionality as well as rapid manufacture and reduced cost ^2^. However, no agreement yet exists on the native phenotype of UC MSCs ^5– 7^, which is an essential pre-requisite for their isolation and minimal expansion.

Prospective isolation of UC MSCs has been attempted after enzymatic digestion followed by selection for specific cell surface markers using fluorescence-activated cell sorting (FACS) or immunomagnetic bead separation techniques. Previous studies showed that CD45-based negative depletion ^5^, neural ganglioside (GD2)- ^7^ and CD146-based positive selection ^6, 8, 9^ could be used to select for clonogenic and multipotential MSC fractions. Although CD146 is expressed on pericytes, which have been proposed as a reservoir of tissue specific progenitors and MSCs ^10, 11^, it is also present on the surface of CD31 ^+^ UC endothelial cells (ECs) ^2, 6, 9^, and therefore cannot be used alone to achieve high-purity of UC MSC preparations.

Whilst the majority of studies in this field used combinations of surface markers to isolate putative MSCs from different tissues, followed by their long-term clonal expansion and analysis of multipotentiality ^12^, another alternative and faster approach could involve measuring the telomere length of freshly-sorted candidate MSC fractions. As immature cells, MSCs by definition should have longer telomeres compared to mature cell types. This idea was first proposed by Flores *et al.* (2008) who showed that stem cell compartments from hair follicles, intestine, testis, cornea and brain are enriched with cells with the longest telomeres ^13^.

The aims of this study were to confirm that CD146 could be a good MSC marker in the UC tissue and to purify candidate MSCs from UC tissue digests based on the non-haemopoietic (CD45 ^-^CD235α ^-^), non-EC (CD31 ^-^), CD146 ^+^ phenotype. We measured telomere lengths in CD146 ^+^ and donor-matched control CD146 ^-^ populations using quantitative real-time PCR (qPCR) and examined the topography of CD146 ^+^ cells *in situ* using immunohistochemistry.

## Materials and methods

### Patients and cells

UC tissue was collected from the UCs of consenting full-term caesarean section patients (n=10). After delivery, UCs were immediately stored in Dulbecco’s phosphate buffered saline (DPBS, #14190-250, Invitrogen, Renfrew, UK) at 4°C. All samples were obtained after written informed consent and the protocols were approved by National Research Ethics Committee (07/Q1205/27). Human primary skin fibroblasts were obtained from Lonza and ATCC (Lonza, Cambridge, UK and ATCC, Middlesex, UK).

### MSC and EC isolation from UC tissue

The whole UC tissue was mechanically dissected in small pieces (~0.2±0.1 g) and washed repeatedly with DPBS followed by enzymatic digestion using 600 U/ml collagenase I (#07902, Stem Cell Technologies, Grenoble, France) ^2^ for 6 hours. Released cells were resuspended in 1:50 v/v DPBS, filtered through a 70 μm cell strainer (#352350, BD Biosciences, Oxford, UK), centrifuged (650 *g*) and counted.

For primary MSC culture, UC tissue digests were seeded in 6-well tissue culture plates (#3516, Corning, Amsterdam, The Netherlands) in non-haematopoietic (NH) expansion medium (#130-091-680, Miltenyi Biotec, Bisley, UK) at a density of 5×10 ^4^ cells/well. After observing 80% cell confluency [denoted passage (p) 0], adherent cells were trypsinised using 0.5% trypsin/EDTA (#15400-054, Invitrogen) and re-seeded at 5×10 ^5^/25 cm ^2^ flask for further passaging which was performed as 1:1 splits until approximately p17. Cultures were fed twice weekly with half media changes. The number and viability of the cells at each passage were evaluated using Trypan blue staining. For primary EC culture, 5×10 ^4^ cells were seeded into 6-well tissue culture plates coated with 2 μg/cm ^2^ fibronectin (#354008, BD Biosciences) in endothelial basal medium (EBM2, #CC-3162, Lonza). On day 2, adherent cells were washed with DPBS and subsequently fed three times per week with half media changes. ECs were passaged using 1:1 splits until p4.

For all cultures, the number of population doublings (PDs) between passages (starting from initial passage; p0) was calculated according to the following equation: PD = log 2 (Nt/Ni), where Ni and Nt are the initial and terminal cell counts, respectively. PDs before p0 were calculated based on colony-forming unit-fibroblast (CFU-F) potential of cells seeded and the number of cells at p0 according to the equation PD = log 2 (N cells at p0/N seeded CFU-Fs). The CFU-F assay was performed in triplicate at the cell seeding density of 5×10 ^4^ cells/well and individual colonies (>50 cells) were counted on day 14 after staining with 1% w/v Crystal Violet (#V5265, Sigma, Hertfordshire, UK).

### Gene expression analysis of UC MSCs

RNA extraction from culture expanded UC MSCs (p4) and fibroblasts was performed using RNA/DNA/Protein purification kit (#23500, Norgen, Ontario, Canada) according to the manufacturer’s instructions and RNA yield was quantified by using NanoDrop 2000 spectrophotometer (Thermo, Essex, UK). Single strand cDNA was synthesised using high-capacity cDNA reverse transcription kit (#4368814, Applied Biosystems, Warrington, UK).

A custom designed 48 gene Taqman low density array (TLDA, Applied Biosystems) contained mesenchymal and endothelial lineage-related transcripts and novel surface receptors that could be used to segregate MSCs from fibroblasts based on previously published data ^14, 15^. To perform TLDA, 200 ng cDNA were used per port. The results were obtained using an ABI PRISM 7900HT SDS (Applied Biosystems). Normalisation of transcript levels relative to reference gene *HPRT* was performed using the formula: 2 ^-ΔCt^, ΔCt=Ct value (selected transcript) - Ct value ( *HPRT*).

### Immunophenotyping of MSC and EC cultures

Phenotypic characterisation was performed on culture expanded MSCs at different passages and on cultured ECs at p4 using: CD31-FITC (#MCA1738F), CD105-PE (#MCA1557PE), CD90-PE (#MCA90PE) (all from Serotec, Kidlington, UK), CD73-PE (#550257), CD146-PE (#550315) (both from BD Pharmingen, Oxford, UK), and CD271-PE (#130-091-885, Miltenyi Biotec). The isotype controls were IgG1-FITC (#550616, BD Pharmingen) and IgG1-PE (#MCA928PE, Serotec). A total of 2×10 ^5^ cells was stained with 5 μl FITC- or PE-conjugated antibodies, and dead cells were excluded using 2 μg/ml propidium iodide (PI, #P1304MP, Invitrogen). Cells were acquired using FACScan equipped with CellQuest software version 3.1 (BD Biosciences) and the proportions of the different fractions were calculated as a percentage of total live cells.

### Fluorescence-activated cell sorting of candidate UC MSCs

Cell sorting was performed using a MoFlo cell sorter equipped with SUMMIT software (Beckman Coulter, Buckinghamshire, UK). Following collagenase digestion of UC tissue, 2×10 ^6^ cells were split into two tubes. One tube was stained with 5 μl of neat CD45-FITC (#F0861), CD235α-FITC (#F0870) (both from DAKO, Cambridge, UK), CD146-PE (BD Pharmingen) and CD31-APC (#130-092-652, Miltenyi Biotec), whereas the other was stained with 2.5 μl of neat isotype controls IgG1-FITC (#550617, BD Pharmingen), IgG1-PE (#MCA928PE, Serotec) and IgG1-APC (#130-098-846, Miltenyi Biotec). After incubation with relevant antibodies and washes, 2 μg/ml 7-aminoactinomycin D (7-AAD) (#A1310, Invitrogen) was added to exclude dead cells before sorting into four fractions: haemopoietic cell fraction (HC), CD45 ^+^CD235α ^+^CD31 ^-^; EC fraction, CD45 ^-^CD235α ^-^CD31 ^+^; candidate MSC fraction, CD45 ^-^CD235α ^-^CD31 ^-^CD146 ^+^ and non-MSC fraction, CD45 ^-^CD235α ^-^CD31 ^-^CD146 ^-^. The latter two subsets were processed for telomere length analysis.

### Telomere length measurements

QIAamp DNA Mini kit (#51306, Qiagen, Crawley, UK) was used for gDNA extraction from cultured MSCs and freshly-sorted CD146 ^+^ and CD146 ^-^ subsets. Samples were run in triplicate using 20 ng gDNA for expanded cells and all gDNA extracted from 1000 cells for the freshly sorted CD146 ^+^ versus CD146 ^-^ subset comparison. Telomere length measurement by SYBR Green qPCR (#4309155, Invitrogen) involved determining the relative ratio of telomere repeat copy number (T) to a single copy gene ( *36B4*) copy number (S): T/S ratio, as previously described ^16, 17^.

### Immunohistochemistry

Immunohistochemistry was used to characterise UC tissue architecture and investigate CD146 cell topography *in situ* to ascertain whether it exhibited the proposed MSC pericyte distribution. Whole UC tissue cross sections were embedded in OCT mounting media (#361603E, VWR, Leicestershire, UK), snap frozen in liquid nitrogen and stored at -80°C. Cryostat sections (6 μm) were mounted on superfrost slides (#48311-703, VWR) and dried overnight at 37°C. Immunohistochemistry was performed using DAKO REAL detection system (#K4065, DAKO) according to the manufacturer’s instructions. Primary antibodies included: CD31 (#CBL468, working concentration 1:10), CD146 (#MAB16985, working concentration 1:50) (both from Chemicon, Watford, UK), CD34 (#M716501, working concentration 1:100), CD271 (#M3507, working concentration 1:20) (both from DAKO), CD90 (#MCA90, working concentration 1:100) and alpha smooth muscle actin (αSMA, #MCA5781GA, working concentration 1:20) (both from Serotec). Antibody binding was visualized using DAKO REAL DAB+ chromogen (#K3468, DAKO) and slides were counterstained by mounting in Harris haematoxylin (#HHS128, Sigma). Slides were mounted using DPX (#317616, Sigma) and images were captured using CAMEDIA C-7070 camera (Olympus, Tokyo, Japan).

### Statistical analysis

The software used for analysis and statistics was GraphPad (GraphPad software, La Jolla, USA). The gene expression results were analysed with Mann-Whitney test for unpaired samples (P<0.05: high significance, 95% confidence interval). Sorted fraction yields were compared using Wilcoxon matched pairs test. The cell sorting and CFU-F results were analysed by Kruskal-Wallis test with Dunn’s correction.

## Results

### Molecular profile of culture-expanded UC MSCs compared to fibroblasts

We initially aimed to confirm the validity of CD146 as a candidate marker of UC MSCs using cultures established following standard MSC protocols ^2^. Mesenchymal tri-potentiality of these cultures was demonstrated in our previous study ^2^. We studied the expression of 45 MSC- and fibroblast-related transcripts by TLDA in these cultures and compared their expression levels to those of negative control skin fibroblasts. Eighteen transcripts were selected ( Table 1) as they showed interesting differences between UC MSCs and fibroblasts. Moreover, five transcripts showed more than two-fold higher expression in UC MSCs. These included *NANOG* (a pluripotency marker), whose expression in UC cultures suggested their greater level of immaturity, as well as *NGFR/*CD271 and *MCAM/*CD146, the markers of native BM MSCs ^18, 19^. Fibroblasts showed stronger expression of eight transcripts ( Table 1).

**Table 1.** Relative gene expression in UC MSCs compared to skin fibroblasts. Normalisation of transcript levels relative to reference gene *HPRT* was performed using the formula: 2 ^-ΔCt^, ΔCt=Ct value (selected transcript) - Ct value ( *HPRT*).

Gene	TaqMan assay ID	Description	UC MSCs	FIBs	UC MSCs to FIBs
***NGFR***	Hs00182120_m1	nerve growth factor receptor	0.11	0.02	5.5
***MCAM***	Hs00174838_m1	melanoma cell adhesion molecule	1.1	0.35	3.14
***SOX9***	Hs00165814_m1	SRY (sex determining region Y)-box 9	0.29	0.12	2.42
***HIF1B***	Hs01121918_m1	aryl hydrocarbon receptor nuclear translocator	2.96	1.23	2.41
***NANOG***	Hs02387400_g1	Nanog homeobox	0.06	0.03	2.0
***HIF1A***	Hs00936371_m1	hypoxia inducible factor 1, α subunit	109.63	58.17	1.88
***LRP5***	Hs00182031_m1	low density lipoprotein receptor related protein 5	7.08	3.91	1.81
***VEGFA***	Hs00900058_m1	vascular endothelial growth factor A	7.01	4.69	1.49
***SFRP4***	Hs00180066_m1	secreted frizzled-related protein 4	0.02	0.03	0.66
***CEBPA***	Hs00269972_s1	CCAAT/enhancer binding protein (C/EBP)	0.03	0.05	0.6
***FRZB***	Hs00173503_m1	frizzled-related protein	0.005	0.01	0.5
***COL1A2***	Hs01028971_m1	collagen, type I, α2	75.61	212.62	0.35
***SPARC***	Hs00277762_m1	osteonectin	28.34	98.73	0.28
***VEGFB***	Hs00173634_m1	vascular endothelial growth factor B	9.52	33.48	0.28
***BMPER***	Hs00403062_m1	BMP binding endothelial regulator	3.06	10.84	0.28
***DDR2***	Hs00178815_m1	discoidin domain receptor tyrosine kinase 2	4.1	17.59	0.23
***FZD4***	Hs00201853_m1	frizzled family receptor 4	0.79	3.64	0.22
***HIF1AN***	Hs00215495_m1	hypoxia inducible factor 1, α subunit inhibitor	3.14	15.21	0.21

### Expression of CD146 and CD271 during extended culture of UC MSCs

**Figure 1.**
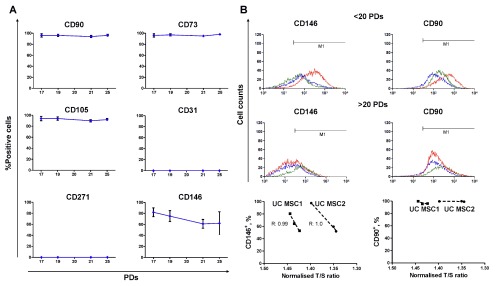
MSC marker expression in culture-expanded UC MSCs. **A** - Surface expression levels of MSC markers (n=3 donors, y error bars indicate SD). **B** - Donor variation of CD146 and CD90 markers in early (<20 PDs) and late (>20 PDs) cultures (M1: marker expression). Bottom panels - telomere T/S ratios were directly correlated to the expression levels of CD146 and not CD90 during culture-expansion.

Having shown *NGFR* and *MCAM* transcript expression in culture-expanded UC MSCs, their surface protein expression at p4 and subsequent passages was next investigated. Phenotypic characterization was performed at four different passages (representing approximately 17, 19, 21, 25 PDs). MSC-specific markers CD90, CD73 and CD105 ^20^ were uniformly positive showing a stable expression profile throughout the expansion, whereas the absence of EC marker CD31 showed no contamination with ECs ( Figure 1A).

Despite the expression of *NGFR* transcript, CD271 surface protein expression was absent in culture-expanded MSCs at all tested time-points ( Figure 1A). CD146 expression declined gradually and correlated with telomere loss in the two cultures tested ( Figures 1A and 1B). This effect was not evident for CD90 which remained stable ( Figure 1B). When comparing the phenotypic profile of UC MSCs with ECs only CD31 revealed high specificity for ECs; CD73, CD105, CD146 were expressed on both UC MSCs and ECs (data not shown).

In combination with the molecular profile of UC MSCs and the previously-published literature ^6, 8, 9^, these data indicated that uncultured UC CD146 ^+^ cells could indeed possess longer telomeres than the remaining CD146 ^-^ cells.

### Telomere length measurement in sorted CD146 ^+^ and CD146 ^-^ populations

**Figure 2.**
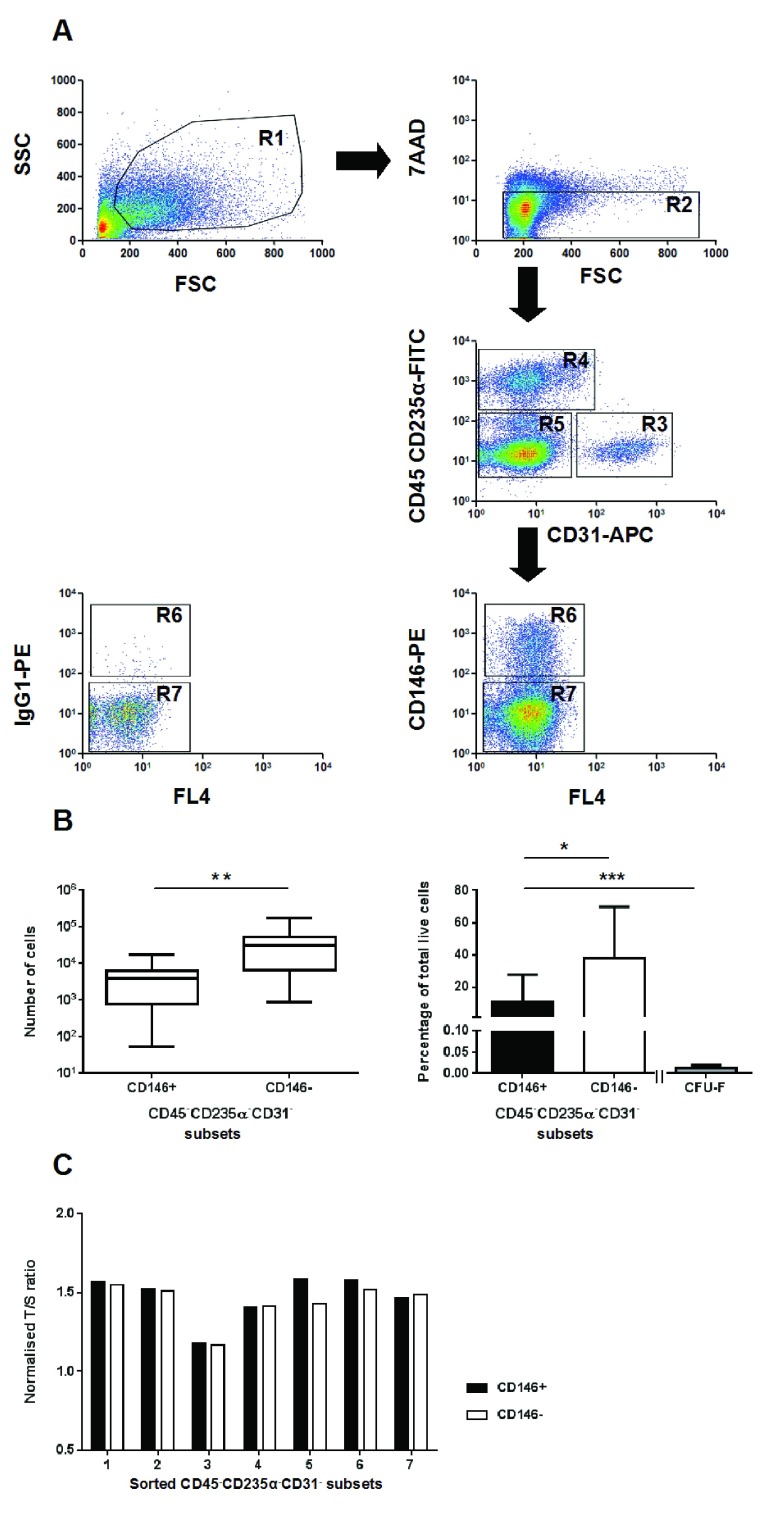
Sorting strategy and telomere length measurements in putative native UC MSCs. **A** - Cell sorting strategy: nucleated cells (upper left graph, R1) were gated based on FSC/SSC profile; live cells (upper right graph, R2) were identified by 7AAD exclusion method. On average, nucleated cells (R1) represented 27% of total events collected whereas excluded events corresponded to red blood cells and cellular debris. Following R2 gating, three distinct populations were evident: middle right graph, R3/ECs, R4/HCs, and R5/double-negative. Gating on double-negative subset (R5) revealed two subsets (bottom right graph), CD146 ^+^ (R6/candidate MSCs) and CD146 ^-^(R7/non MSCs); isotype control staining is shown on the bottom left panel. Cells confined to regions R6 and R7 were sorted and processed for telomere length analysis. **B** - The total yields of CD146 ^+^ and CD146 ^-^ fractions (left panel) and their percentage of total live cells compared to the percentage of CFU-Fs (right panel, n=10 donors for sorted subsets, n=6 donors for CFU-F). Box and whiskers plots represent quartiles and range respectively, bar indicates median, y error bars indicate SD, *p<0.05, **p<0.01, ***p<0.001). **C** - Telomere T/S ratios in sorted subsets, normalised according to Cawthon 2002 (n=7 donors).

The cell sorting strategy for these experiments is described in Figure 2A. Following FSC/SSC gating to define cells (R1), live cells (R2) containing distinct populations of ECs (R3) and HCs (R4) were clearly observed. In general, HCs were most abundant (mean 48% of total live cells, n=10). CD31 ^+^ ECs represented a mean of 1.4% of live cells. Double negative cells (R5) were further subdivided into CD146 ^+^ (R6, candidate UC MSC) and CD146 ^-^ (R7, non-MSC) subsets and sorted into separate RNA lysis buffers. The yields of sorted cell subsets are shown on Figure 2B, left panel.

Within the double-negative cells, the CD146 ^-^ population was predominant over the CD146 ^+^ population (mean 81% and 17% respectively, p<0.05; Figure 2A). When these frequencies were re-calculated in relation to total live cells, the CD146 ^+^ and CD146 ^-^ fractions represented a mean of 10.4% and 37.7%, respectively ( Figure 2B, right panel). This was significantly higher than the frequency of CFU-Fs (as a percentage of total live cells) (mean 0.01%, Figure 2B, right panel). When the telomere length of both sorted subsets (CD146 ^+^ and CD146 ^-^) were tested, the CD146 ^+^ subset exhibited higher telomere lengths compared to the CD146 ^-^ subset in five out of seven donors ( Figure 2C). The median difference suggested that CD146 ^+^ cells’ telomeres were 28bp longer (range 6414–7187bp) compared to CD146 ^-^ cells (range 6393–7120bp); however, the observed differences failed to reach statistical significance. Interestingly, the T/S ratio of the candidate MSC population falls closer to those of the adult tissues (BM MSCs and endometrial cells) than foetal tissues described by Guillot *et al.* ^21^.

The high frequency of CD45 ^-^CD31 ^-^CD235α ^-^CD146 ^+^ cells compared to the CFU-F frequency and the lack of consistent and significant enrichment for cells with long telomeres indicated that the CD146 ^+^ UC fraction most likely contained non-haemopoietic cells with varying degrees of maturity.

### CD146 expression in UC tissue *in situ*

**Figure 3.**
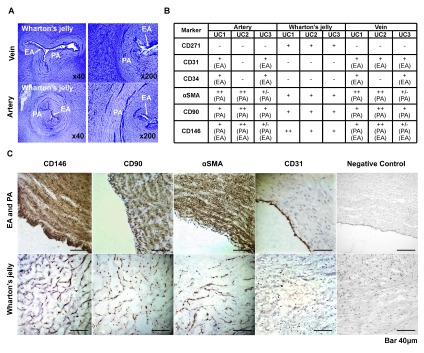
Tissue architecture of UC vein, artery and surrounding Wharton’s jelly. **A** - Images show endothelial area (EA, indicated by arrows), perivascular area (PA, multiple layers of muscle fibres) and Wharton’s jelly area. **B** - Expression of MSC and EC markers in UC tissue. **C** - Staining of MSC and EC markers in UC tissue (representative donor and cross sections; (+) symbol indicates the expression of a marker and (-) symbol indicates the absence of expression of a marker).

Initial haematoxylin staining ( Figure 3A) revealed the basic structure of the UC tissue where the UC vein and artery could be seen. This was surrounded by the single thickness endothelial area (EA) and highly organised muscular perivascular area (PA). The Wharton’s jelly (WJ) matrix could also be seen spanning intra-muscular areas.

Immunohistochemistry was next used to investigate marker expression *in situ* including semi-quantitative assessment in different anatomical areas ( Figures 3B and 3C). Three optical microscope fields (×200) were evaluated per anatomical area of UC tissue. CD271 was present at low levels in the WJ area only. EC-specific CD31 and CD34 were present in the EA of the vessels. The intracellular marker of smooth muscle cells; αSMA ^22^ was expressed and had its highest positivity in the PA surrounding vessels. CD90 and CD146 were highly expressed in most UC compartments, including the PA; however CD146 but not CD90 showed positivity for the EA area. This was consistent with previously published literature ^9^. Representative photomicrographs are shown in Figure 3C.

Overall, our immunohistochemistry results revealed the expected topography of CD31 ^+^ and CD34 ^+^ ECs, the expression of CD271 in the WJ area, and the preferential topography of CD90 and αSMA in PA. Consistent with previous findings ^9^, CD146 was expressed in WJ, PA and EA, with the highest proportion of cells present in the PA. Wide distribution of CD146 ^+^ cells in all anatomical areas of UC tissue was consistent with the high frequency of CD45 ^-^CD31 ^-^CD235α ^-^CD146 ^+^ cells evident by flow cytometry. This indicated that UC MSC isolation alone, even after the removal of CD31 ^+^ ECs, was not sufficient to purify native MSCs from the UC tissue.

Version 2. CD146-based cell sorting and telomere length in umbilical cord UC MSC and fibroblast cultures were evaluated for their expression levels of selected transcripts characterizing multiple cell fates *in vivo*. Ct values obtained using Taqman qPCR technology are shown in dataset a (UC=Umbilical cord; FIB=fibroblast cell lines). The flow cytometry phenotype profiles of UC MSCs (cultures tested against a panel of MSC markers) and UC ECs (cultures tested against a panel of EC markers) are shown in dataset b. Telomere lengths of cultured UC MSC1 and MSC2 samples are displayed in dataset c (telomere lengths calculated as T/S ratio in different passages of UC MCS cultures). Telomere lengths as T/S ratio and calculated length of freshly sorted cells are shown in dataset d (Telomere length was calculated as T/S ratio and length in bp from the freshly sorted CD45 ^-^CD235α ^-^CD31 ^-^CD146 ^+^ (candidate MSCs) and donor-matched CD45 ^-^CD235α ^-^CD31 ^-^CD146 ^-^ (non-MSCs) of n=7 UCs).

## Discussion

The present study assessed the possibility of using CD146-based cell sorting and telomere length analysis for establishing the identity of mesenchymal stem cells in human UC. Previous studies have shown that the UC CD146 ^+^ subset contained MSCs able to differentiate into osteoblasts, chondrocytes and adipocytes ^8, 9^. Other studies have shown that ‘true’ immature stem cells have much longer telomeres compared to the remaining mature cells ^13, 23^. Here we tested whether CD146 ^+^ UC cells, that were depleted of contaminating haemopoietic and endothelial cells, had longer telomeres compared to the corresponding CD146 ^-^ population.

Initially we performed gene expression analysis, flow cytometry and telomere length measurements on culture-expanded UC MSCs during their extensive passaging. The mesenchymal tripotentiality of these cultures has been demonstrated in our previous study ^2^. In this study, gene expression analysis revealed that *MCAM*/CD146 was expressed at higher levels (>3-fold) in UC MSCs compared to fibroblasts, confirming its potential specificity for MSCs. Whereas common MSC-specific markers CD73, CD105, CD90 ^20^ displayed stable expression throughout passaging, CD146 expression declined with the increased number of cell divisions at later passages consistent with a loss of multipotential progenitors ^4^. The CD271 surface marker was not expressed at any stage during UC MSC cultivation, consistent with the loss observed in BM MSCs ^14, 24, 25^. Although CD271 is expressed on uncultured BM MSCs, it was not selective for UC blood MSCs ^19, 26^. Therefore, CD271-based MSC isolation from UC tissue was not pursued further. However, the decrease in CD146 during expansion correlated with telomere erosion in the same cultures, supporting the idea that CD146 could mark the most immature cells *in vivo*. Based on the present findings and previous data ^9^, CD146 was selected as a candidate positive marker for sorting native MSCs from UC digests. CD45/CD235α and CD31 were used to exclude native HCs and ECs from the analysis, respectively ^2^.

Sorted CD146 ^+^ and CD146 ^-^ subsets were next compared with respect to their telomere length. Several previous studies provided initial evidence that UC MSCs express telomerase continuously and hence were able to retain long telomeres ^27, 28^. In UC blood MSCs, a distinct SSEA-4 ^+^CD105 ^+^MSCA ^-^1 ^-^CD90 ^-^ cell population was shown to have longer telomeres than the SSEA-4 ^-^CD105 ^+^MSCA ^-^1 ^+^CD90 ^+^ subset ^29^. Although evident in five out of seven experiments in our study, there was no significance to the difference in telomere length between CD146 ^+^ and CD146 ^-^ subsets potentially indicating contamination of MSCs with more mature cells in the CD146 ^+^ subset. The high frequency of CD146 ^+^ cells (10.4%) was in stark contrast to the CFU-F frequency (~0.01%), which was consistent with previous findings ^5, 30^. Furthermore, the immunohistochemistry data confirmed the broad reactivity of CD146 with UC tissue anatomical areas ^9^. Altogether, these data provided the first indication that the CD146 ^+^ population was unlikely to represent pure native UC MSCs.

Alternatively, a lack of significant difference in telomere lengths could be explained by the limited sensitivity of qPCR assay to measure telomere lengths ^31^. Telomere length assessment in the majority of studies using cultured MSC is based on terminal restriction fragment (TRF) analysis ^32– 36^, which has the disadvantage of producing a smear of bands rather than a discrete band/point affecting accurate quantifications. Additionally, the large number of cells required for such analysis precluded its use on sorted UC cells. On the other hand, studies investigating the correlations between telomere lengths derived by qPCR and TRF indicated good levels of correlation (r>0.823) ^37^, although qPCR was shown to be limited in its ability to measure the longest telomeres ^31^. This potential technical limitation of qPCR, as well as working at the lower limit of DNA concentrations, could have affected the accuracy of the telomere analysis in the present study.

Our findings could be further compounded by the large donor-to-donor variation, which was evident in culture expanded UC MSCs (~10% between donor 1 and 2, Figure 1B), but also with sorted subsets ( Figure 2C). One recent study has demonstrated that telomere lengths could be heritable, with the parental age at conception being a factor affecting offsprings’ telomere length in leukocytes (LTL) ^37^. Although the mode of LTL inheritance has been suggested to be X-linked ^38^, another study shows a paternal mode of heritability ^39^. The parental ages of the UC donors were not known/recorded in this study but would be an interesting subject for the future work. Previous studies have suggested that the magnitude of inter-individual variation in telomere lengths could exceed the variation between cell types within the same individual ^40– 42^, therefore telomere length measurement approaches should be further refined before they can be used as a tool to identify MSCs *in vivo*.

To conclude, the broad availability of UC tissue makes it amenable to be used in cell therapy and regenerative medicine interventions. To this end, the present study showed that in the UC, CD146 ^+^ cells were too numerous to be selective for pure native MSCs and were likely to contain more mature cells. Therefore, additional markers would be needed to isolate MSCs from UC tissue.

## Data availability

*F1000Research*: Dataset 1. Version 2. CD146-based cell sorting and telomere length in umbilical cord, http://dx.doi.org/10.5256/f1000research.4260.d34848 ^43^

## Consent

Written informed consent has been obtained from full-term caesarean section patients for the use of clinical samples in the present study.
