# Supplementary material for: The assessment of CD146-based cell sorting and telomere length analysis for establishing the identity of mesenchymal stem cells in human umbilical cord
Source: F1000Res. 2014 Aug 27;3:126. Originally published 2014 Jun 11. [Version 2] doi: 10.12688/f1000research.4260.2 (PMC4162508; doi:10.12688/f1000research.4260.2)
Supplement: Peer review history

# Peer review: Referee response for version 2

**DOI:** 10.5256/f1000research.5485.r5969

**Published:** 2014-08-28

**Authors:** Simone Pacini

**Affiliations:** 1 Department of Oncology, Transplants and New Advances in Medicine, University of Pisa, Pisa, Italy

Authors addressed any queries and the revised manuscript clarifies all the aspects that were originally ambiguous, in the first version of the paper. Thus I believe that the manuscript, in this form, fulfils the scientific standard requirements.

I have read this submission. I believe that I have an appropriate level of expertise to confirm that it is of an acceptable scientific standard.

# Peer review: Referee response for version 1

**DOI:** 10.5256/f1000research.4561.r5441

**Published:** 2014-07-23

**Authors:** Simone Pacini

**Affiliations:** 1 Department of Oncology, Transplants and New Advances in Medicine, University of Pisa, Pisa, Italy

I carefully read the manuscript entitled “ *The assessment of CD146-based cell sorting and telomere length analysis for establishing the identity of mesenchymal stem cells in human umbilical cord*” from Kouropis *et al.*  The aim of the study is verifying the hypothesis that umbilical cord-derived MSCs reside in the CD146-positive fraction, and could be discriminated by their possible longer telomeres. The authors demonstrated that frequency of CD146-positive cells in the culture-initiating suspension was much higher than the frequency of CFU-F  confirming that this population also include non MSC-progenitors. Telomere length analysis revealed non-significant differences between CD146-positive and –negative cells, in three of four samples. Thus, the authors correctly conclude that the CD146-positive population was unlikely representing pure native UC-MSCs, and that a prospective isolation of *ex vivo* MSC necessarily should include more antigens to investigate.

My review will be concentrated on some technical aspects regarding conduction of cell sorting and telomere analysis, which should be clarified. My major comments are as follows:

 

- From the density plots reported in figure 1A, it is not clear if a morphology gate was applied in the SSC/FSC plot, to exclude events of no-cellular origins. A Gate was applied in the dead-cells exclusion plot and in R1 would be counted no-cellular 7-AAD negative events, which could significantly alter the CD146 positive or negative percentages on total living cells. The authors extensively discussed the percentage of CD146-positive frequency in correlation to CFU-F frequency. Thus, the authors should clarify this aspect, and I strongly suggest clearing the flow cytometry data from the non-cellular events.
- As the CD146-positive and -negative populations are not sufficiently separated in the R4 gate, the sorting gates R5 and R6 should be defined by applying isotypic control. Moreover, I suggest reporting the isotypic control plot in the figure, in order to demonstrate the absence of events in the R5 region.
- Purity, and possible Yield, of sorted cells should be indicated in the results section, in order to reassure the reader of the quality of the sorting process.
- Telomere length has been described as T/S ratio, reported data are close to the value of 1.0 and two of four have values lower than 1.0. How could it be possible to detect telomere repeat copy numbers lower than the single copy gene (36B4)? According to Cawthon R.M. (2002) T/S ratios should be normalized. PCR efficiency could vary gene to gene, and should be evaluated in order to normalize qPCR data with the efficiency correction factor. 

I have read this submission. I believe that I have an appropriate level of expertise to confirm that it is of an acceptable scientific standard, however I have significant reservations, as outlined above.

# Response

**Published:** 2014-08-21

**Authors:** Dimitrios Kouroupis

**Affiliations:** University of Leeds, UK

- *“From the density plots reported in figure 1A, it is not clear if a morphology gate was applied in the SSC/FSC plot, to exclude events of no-cellular origins. A Gate was applied in the dead-cells exclusion plot and in R1 would be counted no-cellular 7-AAD negative events, which could significantly alter the CD146 positive or negative percentages on total living cells. The authors extensively discussed the percentage of CD146-positive frequency in correlation to CFU-F frequency. Thus, the authors should clarify this aspect, and I strongly suggest clearing the flow cytometry data from the non-cellular events.” *
  We are very grateful to Dr Pacini for his careful reading of our work and for his insightful comments. We believe that Figure 1A that he refers to is in fact Figure 2A as this pertains to our cell sorting experiments from umbilical cord (UC) tissue digests. We can confirm that we did perform FSC/SSC gating to remove anuclear red cells and cellular debris in all our sorting experiments. It was an oversight that this was omitted in the original submission and we thank the reviewer for highlighting this. We have now modified the relevant section of our Materials and Methods as well as Figure 2A to reflect this (gate R1). Additionally, we have added average percentage of cells within R1 out of all events collected into the appropriate Figure Legend.

 

- *“As the CD146-positive and -negative populations are not sufficiently separated in the R4 gate, the sorting gates R5 and R6 should be defined by applying isotypic control. Moreover, I suggest reporting the isotypic control plot in the figure, in order to demonstrate the absence of events in the R5 region. Response: We appreciate this comment. In our experiments with UC tissue digests, a distinct CD45-CD235a-CD31-CD146+ population was always evident above the isotype control staining.” *
  We further acknowledge that our original density plots failed to show that our sorting gates were always set apart to ensure maximal sorting purity. We have made new flow cytometry plots for panel 2A to reflect all of these changes (FSC/SSC gating and isotype control panel).

 

- *“Purity, and possible Yield, of sorted cells should be indicated in the results section, in order to reassure the reader of the quality of the sorting process.”*
  We have now provided information on CD146+ and CD146- fractions’ cell yields in Figure 2B, left panel. We acknowledge that we did not make clear in our original submission how many sorting experiments we performed in total, we confirm that this is ten for the sorting experiments. Fraction purities post-sort are always >95% using our cell sorter (see Churchman *et al*., 2012).

 

- *“Telomere length has been described as T/S ratio, reported data are close to the value of 1.0 and two of four have values lower than 1.0. How could it be possible to detect telomere repeat copy numbers lower than the single copy gene (36B4)? According to* *Cawthon R.M. (2002)* *T/S ratios should be normalized. PCR efficiency could vary gene to gene, and should be evaluated in order to normalize qPCR data with the efficiency correction factor.”*
  We appreciate this comment and agree that it is indeed a valid point that we should clarify further. Our method was based upon that of Cawthon R.M. (2002) as stated above and in the text, however when using unexpanded cells sorted from UC the cell numbers were limited because of the amount of tissue processed and inevitable cell losses caused by multiple centrifugation steps prior to cell sorting. Due to this we elected to standardise our telomere length experiments to 1000 sorted cells per fraction (previously overlooked from methods). We accept that we were working at the lower limit of DNA concentrations (acknowledgement of this is now added as a new sentence in Discussion), but believe that with triplicate replicates and extremely comparable standard curves (prepared using immortal cell lines) on each plate for inter-plate comparisons, our work is technically sound, and that our standardisation and technical control was suitable for comparing the differences between the CD146+ and CD146- populations. We did not normalise our T/S ratios and chose to show the data as a direct comparison of sample pairs and therefore some of these values fell below 1. This was also observed in the Cawthon paper; a ‘raw’ T/S ratio of 0.69 is what he normalised against to give the lowest value of 1 (Figure legend 5). Values below 1 can also be observed in Guillot *et al.* (2007).We have added an interesting observation that our umbilical cord T/S ratios fall closer to the adult tissues than the foetal tissues in that study. We now present normalized T/S ratios adjusted according to Cawthon R.M. (2002) in Figure 2C and conversion to telomere length in the text. We have also strengthened this figure with new data from a further 3 sorting experiments (new total n=7) and have performed statistical analysis. The CD146+ fraction possesses telomeres with median value 28bp longer than the CD146- fraction, although this was not significant (Wilcoxon).

# Peer review: Referee response for version 1

**DOI:** 10.5256/f1000research.4561.r5083

**Published:** 2014-07-04

**Authors:** Armin Attar

**Affiliations:** 1 Shiraz University of Medical Sciences, Shiraz, Iran

In the current study, the investigators have used cell sorting techniques as well as telomere length analysis to assess the identity of UC tissue derived mesenchymal stromal cells. The manuscript is scientifically sound and deserves indexation.

I have read this submission. I believe that I have an appropriate level of expertise to confirm that it is of an acceptable scientific standard.
